# Deletion of *TMEM268* inhibits growth of gastric cancer cells by downregulating the ITGB4 signaling pathway

**DOI:** 10.1038/s41418-018-0223-3

**Published:** 2018-10-25

**Authors:** Dubeiqi Hong, Xuan Zhang, Riyong Li, Jiahong Yu, Yaxin Lou, Qihua He, Xuanze Li, Dong Xu, Ping Lv, Jian Lin, Yingyu Chen

**Affiliations:** 1 Department of Immunology, Peking University School of Basic Medical Sciences, Beijing, China; 2 NHC Key Laboratory of Medical Immunology, Peking University, Beijing, China; 3 Center for Human Disease Genomics, Peking University, Beijing, China; 4 Medical and Healthy Analytical Center, Peking University, Beijing, China; 5 Key Laboratory of Photochemical Conversion and Optoelectronic Materials, Technical Institute of Physics and Chemistry, Chinese Academy of Sciences, Beijing, China; 6 Department of Clinical Laboratory, Peking University First Hospital, Beijing, China; 7 College of Chemistry and Molecular Engineering, Innovation Center for Genomics, Peking University, Beijing, China

**Keywords:** Tumour-suppressor proteins, Integrins

## Abstract

Transmembrane protein 268 (*TMEM268*) encodes a novel human protein of previously unknown function. This study analyzed the biological activities and molecular mechanisms of TMEM268 in vivo and in vitro. We found that *TMEM268* deletion decreases cell viability, proliferation, and cell adhesion as well as causing S-phase cell cycle arrest and disrupts cytoskeleton remolding. Xenograft tumor mouse model studies showed that *TMEM268* deletion inhibits the tumorigenesis of BGC823 gastric cancer cells. In addition, *TMEM268-*deleted BGC823 cells failed to colonize the lungs after intravenous injection and to form metastatic engraftment in the peritoneum. Molecular mechanism studies showed a C-terminal interaction between TMEM268 and integrin subunit β4 (ITGB4). *TMEM268* knockout promotes ITGB4 ubiquitin-mediated degradation, increasing the instability of ITGB4 and filamin A (FLNA). The reduced ITGB4 protein levels result in the disassociation of the ITGB4/PLEC complex and cytoskeleton remodeling. This study for the first time demonstrates that TMEM268 plays a positive role in the regulation of ITGB4 homeostasis. The above results may provide a new perspective that targeting the TMEM268/ITGB4 signaling axis for the treatment of gastric cancer, which deserves further investigation in the future.

## Introduction

Integrins are transmembrane glycoproteins that form an important family of heterodimeric cell adhesion receptors [1]. They have been shown to be critical in controlling cell−cell and cell−matrix interactions, and in connecting the extracellular matrix (ECM) to the cell cytoskeleton. They exert diverse effects on the proliferation, migration, and invasion of cancer cells [2–5], and regulate differentiation, angiogenesis, epithelial-to-mesenchymal transition, and even therapeutic outcomes [6–8]. These properties make integrins attractive prognostic indicators and therapeutic targets for cancer [9, 10].

The integrin subunit β4 (ITGB4) heterodimerizes with the integrin subunit α6 (ITGA6) to form a receptor for laminin (LN). ITGB4 has an unusually long cytoplasmic domain that has distinctive cytoskeletal and signaling functions [11]. One fundamental function of integrin α6β4 in polarized epithelial cells is to form a stable attachment to the basal membrane through the formation of hemidesmosomes. It is linked to the actin cytoskeleton. In vitro and in vivo experiments indicate that ITGB4 promotes tumorigenesis and metastasis in different types of cancers [12, 13]. Increasing evidence indicates that the overexpression of ITGB4 is correlated with an aggressive phenotype and poor prognosis in breast cancer, lung cancer, pancreatic cancer, cervical cancer, and gastric cancer [14–18]. These observations suggest that expression of ITGB4 confers an evolutionary advantage during tumor progression and potential therapeutic target. The regulatory mechanism for the expression of ITGB4 protein remains to be clarified.

As part of a human genomics project, we cloned hundreds of functionally unknown human open-reading frames (ORFs) by searching the human RefSeq and expressed sequence tag databases in GenBank. Using a cell-based high-throughput assay, we identified several novel genes associated with cell viability, including transmembrane protein 268 (*TMEM268*), previously identified as *C9orf91* [19]. Transcription of the human *TMEM268* gene produces two experimental confirmed mRNAs (*TMEM268-v1* and *TMEM268-v2*), both of which encompass nine exons and eight introns. The full length of human *TMEM268-v1* cDNA is 4413 base pairs (bps) comprising an ORF encoding a predicted 37.6 kDa protein of 342 amino acids. This TMEM268-v1 has been chosen as the canonical sequence, usually abbreviated as TMEM268. The full-length of *TMEM268-v2* cDNA is 4481 bps long, its ORF encodes a predicted 37.7 kDa protein of 343 amino acids. The difference between the amino acid sequences of TMEM268-V1 and TMEM268-V2 is that the latter has an extra Glutamine (Q) behind 71 Isoleucine (I) (71: I → IQ), and all the other amino acids are the same (https://www.uniprot.org/uniprot/Q5VZI3). Transmembrane analysis (www.cbs.dtu.dk/services/TMHMM-2.0/) suggests that TMEM268 has two conserved TM domains (amino acids 104–126 and 130–152) and a domain of unknown function (DUF4481, amino acids 38–328) [20]. To our knowledge, no functional studies have been performed on this protein.

In the present study, we demonstrate that *TMEM268* deficiency in gastric cancer cells inhibits cell growth, adhesion, and causes cell cycle arrest. Mechanistically, TMEM268 interacts with ITGB4; *TMEM268* deletion promotes ITGB4 degradation via the protease pathway. Additionally, deletion of *TMEM268* facilitates the disintegration of ITGB4 and Plectin, impairs FLNA stability and the F-actin network, which eventually leads to cytoskeletal remolding in cancer cells.

## Results

### Inactivation of *TMEM268* inhibits cell growth and reduces tumorigenesis in gastric cancers cells

Data from RT-PCR and western blotting proved that TMEM68 is expressed in many human cell lines (Fig. S1A and B). Immunofluorescence assay demonstrated that the TMEM268 protein was mainly present in the endoplasmic reticulum and plasma membrane (Fig. S2). These data are consistent with data reported in The Human Protein Atlas for TMEM268 (http://www.proteinatlas.org/ENSG00000157693-TMEM268).

To clarify the physiological role of TMEM268, we conducted a series of experiments in *TMEM268*-depleted cells. We first identified an effective *shRNA* against *TMEM268* in BGC823 and SGC7901 cell lines (Fig. S3A). MTS assay showed that cell viability of *shTMEM268*-transfected cells is significantly lower than that in the *shControl* group (Fig. S3B and C). A 5-ethynyl-2′-deoxyuridine (EdU) incorporation assay demonstrated that *shTMEM268*-transfected cells contain lower percentage of EdU-positive cells than control cells (Fig. S3D and E). This antiproliferative effect was further demonstrated in a colony formation assay (Fig. S3F and G).

**Fig. 1:**
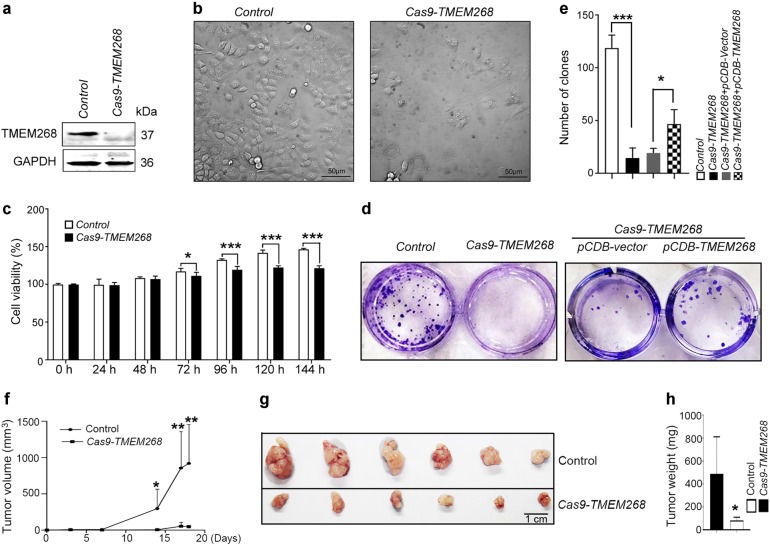
*TMEM268* knockout inhibits cell growth and reduces tumorigenicity. **a** Western blot analysis of TMEM268 expression in control cells (*TMEM268* WT) and *Cas9-TMEM268*/BGC823 cells. **b** *Control* and Cas9-TMEM268/BGC823 cells were seeded in six-well plates (1×10^5^ cells/well). Seventy-two hours later, representative images were obtained by optical microscopy. **c** *Control* and *Cas9-TMEM268*/BGC823 cells were seeded in 96-well plates (3×10^3^ cells/well; five replicates), serum-starved for 18 h and then pulsed with 10% FCS for different lengths of time. Cell viability was detected by MTS assay. Data represent the mean ± SD of three independent experiments. **d** Representative images of colony formation by indicated cells. **e** Number of clones counted in three independent experiments. Data are expressed as mean ± SD. *P < 0.05, ***P < 0.001. **f** The *Control* or *Cas9-TMEM268/*BGC823 cells were injected subcutaneously in BALB/c nude mice. Development of tumors (mean volume ± SD) was monitored using calipers. **g**, **h** Excised xenograft tumors were imaged and weighed on day 19. *P < 0.05, **P < 0.01, ***P < 0.001

To further prove the function of TMEM268 in gastric cancer cells, we knocked out *TMEM268* in BGC823 cells. Through a series of screenings, a *Cas9-TMEM268/BGC823* clone was selected. Sequence analysis revealed that the selected clone contained a 4 bp deletion (ACAATG → TG) resulting in a frame shift which disrupts the *TMEM268* ORF, leading to deletion of the TM domains and C-terminal (Fig. S4). Western blotting confirmed that the TMEM268 protein was not detectable in *Cas9-TMEM268/*BGC823 cells (Fig. 1a). It was found that *Cas9-TMEM268*/BGC823 cells displayed a slow cell growth, accompanied by cell flattening, as well as detachment from the culture dish (Fig. 1b). The growth arrest of *Cas9-TMEM268*/BGC823 cells was determined by MTS assay (Fig. 1c) and colony formation assay (Fig. 1d, e). The specific effects of *TMEM268* knockout were assessed in a rescue experiment. As shown in Fig. 1d, e, overexpression of TMEM268 in *Cas9-TMEM268/*BGC823 cells significantly increases the number of clones compared to pCDB-vector transfected cells. These results further confirm that knockout of *TMEM268* inhibits growth of gastric cancer cells.

The in vivo effects of TMEM268 were evaluated using a gastric cancer xenograft model established in BALB/C nude mice. These mice were subcutaneously injected with *TMEM268* wild-type BGC823 cells or *Cas9-TMEM268/*BGC823 cells. The tumor growth curves are shown in Fig. 1f. The *Control* group developed grossly visible tumors at the site of injection. By comparison, the *Cas9-TMEM268* group displayed smaller tumors. The tumor weights in the *Cas9-TMEM268* group are markedly lighter than those of the *Control* group (Fig. 1g, h). Collectively, these data indicate that the inactivation of *TMEM268* inhibits cell proliferation in gastric cancer cells.

### *TMEM268* knockout causes S-phase cell cycle arrest

We next analyze whether the growth arrest induced by *TMEM268* loss is mediated by apoptosis. Data from flow cytometry analysis indicated that the apoptotic cells were not significantly different between *Control* and *Cas9-TMEM268*/BGC823 cells (Fig. S5), suggesting that the decreased viability of *Cas9-TMEM268*/BGC823 cells is not related to apoptosis.

**Fig. 2:**
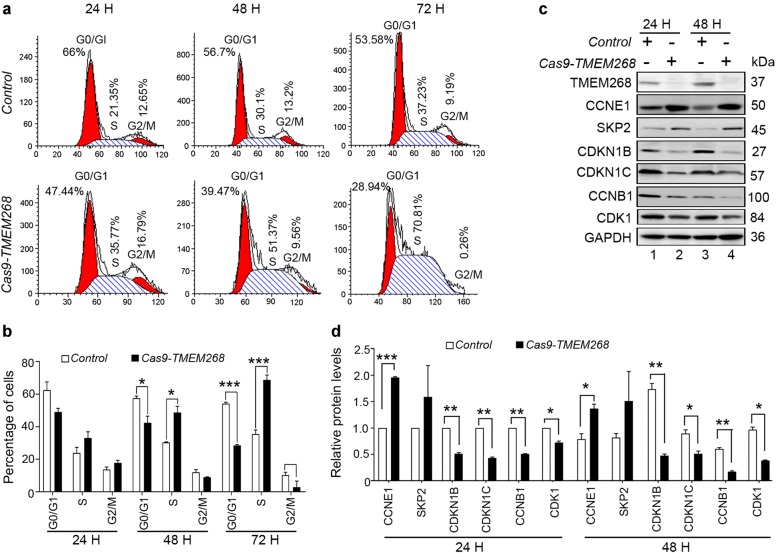
*TMEM268* knockout causes S-phase cell cycle arrest. **a** *Control* and *CAS9-TMEM268*/BGC823 cells were serum-starved for 24 h and then pulsed with 10% FCS for different amounts of time. Cell cycle distribution was assayed by flow cytometry. **b** Percentages of G0/G1, S, and G2/M phase cells. Each bar represents the mean ± SD of three independent experiments. **c** Cells were treated as described in (**a**), and protein levels detected by western blotting. **d** Quantification of the levels of the indicated proteins relative to GAPDH in cells treated as described in (**a**). Average value for control cells (24 h) was normalized to 1. Data shown represent the mean ± SD of three independent experiments. *P < 0.05, **P < 0.01, ***P < 0.001

We further explored the effect of TMEM268 on cell cycle progression. As shown in Fig. 2a, b, a much higher percentage of cells in S phase is observed in *Cas9-TMEM268/*BGC823 cells than in the *Control* group. In each case, there is a concomitant reduction in the proportion of cells in the G0/G1 and G2/M phases.

Cell cycle progression depends on the sequential expression of stage-specific cyclins and the activity of their corresponding cyclin-dependent kinases (CDKs) [21]. G1/S transition is required for the activity of the cyclin E1 (CCNE1)/CDK2 complex. CCNE1 protein accumulates at the G1/S-phase boundary and is degraded as cells progress through S phase, triggering G1 → S transition [22]. CDK inhibitor 1B (CDKN1B) and CDK inhibitor 1C (CCKN1C) bind to and prevent the activation of the CCNE1/CDK2 complex, thus controlling cell cycle progression at G1 [23]. S-phase kinase-associated protein 2 (SKP2) regulates the S-phase entry of cells by inducing the degradation of the CDK inhibitors CDKN1B and CDKN1C [24]. The cyclin B1 (CCNB1)/CDK1 complex is essential for S/G2 and G2/M phase cell cycle transitions [25]. In accordance with the observed S-phase arrest, we found that knockout of *TMEM268* increased the expression of CCNE1 and SKP2 and decreased the levels of CDKN1B and CCKN1C (Fig. 2c, d), allowing the cells to progress from G1 to the S phase. The levels of CCNB1 and CDK1 were also downregulated in *Cas9-TMEM268/*BGC823 cells (Fig. 2c, d), thus inhibiting S/G2 and G2/M transitions. Collectively, these data suggest that *TMEM268* knockout induces S-phase arrest resulting from enhanced initiation and progression from G1 to S phase and the concomitant inhibition of the S to G2/M checkpoint through inactivation of the CCNB1/CDK1 complex.

### *TMEM268* knockout impairs cell adhesion

To examine the effects of endogenous TMEM268 on cell migration, a Transwell assay was performed in *Cas9-TMEM268*/BGC823 and *Control* cells. The results revealed that the number of cells adhered to the membrane is significantly decreased in the *Cas9-TMEM268* group compared with the *Control* group (Fig. S6A and B). However, many cells were observed at the well bottom and in suspension in *Cas9-TMEM268* group, few cells were observed in the *Control* group. Statistical analysis did not show any differences in total cell number between *Cas9-TMEM268* group and *Control* group, which includes membrane-adhered cells and the shedding cells (Fig. S6C). These data suggest that inactivation of *TMEM268* may not affect cell migration in vitro, it reflects a decrease of cell adhesion.

**Fig. 3:**
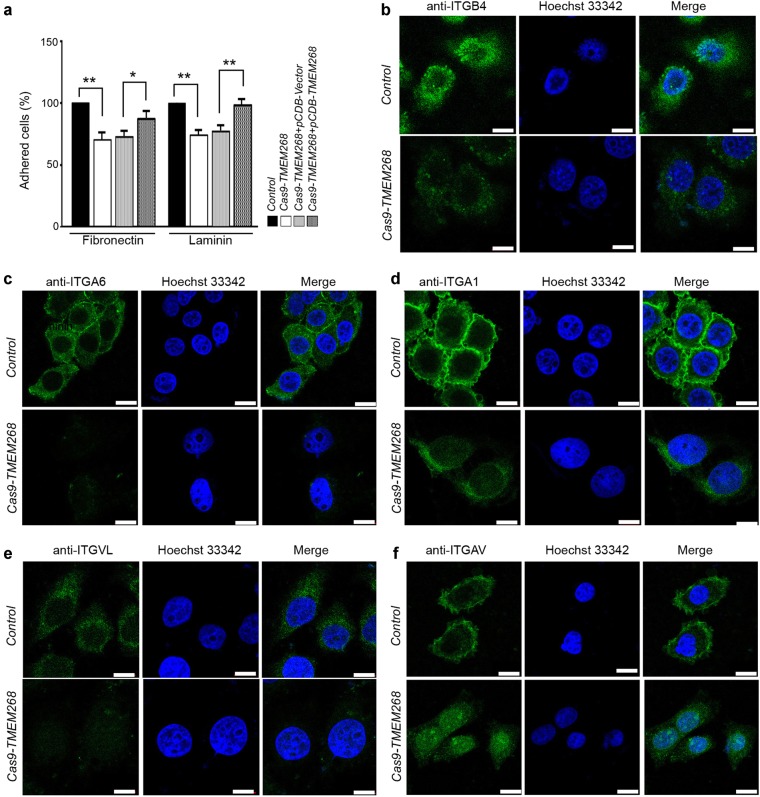
*TMEM268* knockout decreases cell adhesion. **a** *Control* and *Cas9-TMEM268*/BGC823 cells were allowed to attach to 96-well plates precoated with fibronectin or laminin. The unadhered cells were removed at 30 min, and the number of adhered cells was determined using an MTS assay (*P<0.05, **P<0.01). **b**−**f** *Control* and *Cas9-TMEM268*/BGC823 cells were plated on glass slides for 24 h, incubated with the indicated FITC-conjugated antibodies. Nuclei were stained with Hoechst 33342. Representative images obtained from confocal microscopy are shown. Scale bars, 10 μm

We next explored the effect of *TMEM268* deficiency on cell adhesion ability. As shown in Fig. 3a, compared with the control group, the ability of *Cas9-TMEM268/*BGC823 cells to adhere to laminin (LN) and fibronectin (FN) is decreased, indicating a decrease in cell−ECM adhesion. Data from rescue experiments show that overexpression of TMEM268 in *Cas9-TMEM268*/BGC823 cells significantly increases cell−LN and cell–FN adhesion compared with the pCDB-Vector group, demonstrating the rescue of cell−ECM adhesion.

We further analyzed adhesion molecules on the cell membrane associated with cell−matrix interactions. It was observed that in *Cas9-TMEM268/*BGC823 cells, the ITGB4 fluorescence signal on the surface of the cell membrane was significantly downregulated compared to *Control* cells (Fig. 3b), while the fluorescence signal of ITGA6 can hardly be detected (Fig. 3c). The membrane fluorescence of integrin subunit alpha 1 (ITGA1) and integrin subunit alpha L (ITGAL) was also decreased in *Cas9-TMEM268/*BGC823 cells (Figs. 3d, e). The fluorescence of integrin subunit alpha V (ITGAV) could not be detected on the cell surface in *Cas9-TMEM268/*BGC823 cells (Fig. 3f), but intracellular fluorescence signal was observed, suggesting that the internalization of ITGAV might occur. Collectively, our data suggest that *TMEM268* inactivation may inhibit cell adhesion ability by downregulating adhesion molecules on the cell surface.

### TMEM268 associates with ITGB4 via their C-terminals, and *TMEM268* deletion promotes ITGB4 ubiquitination and degradation

**Fig. 4:**
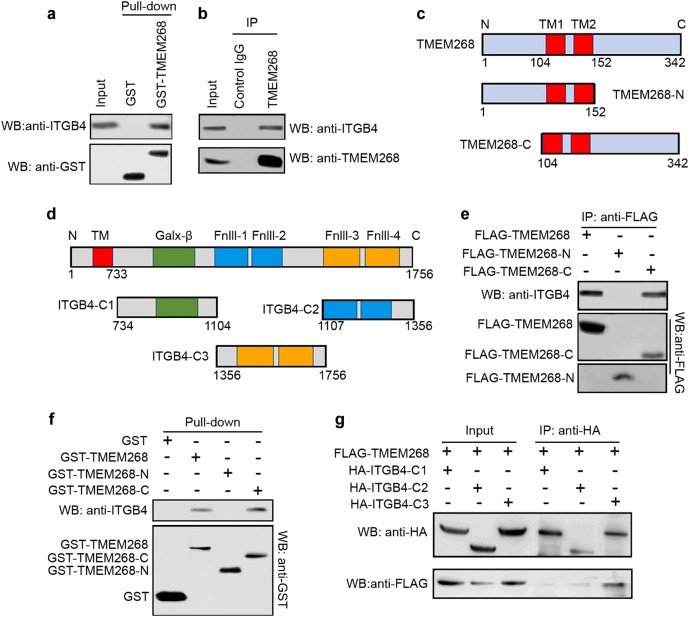
The TMEM268/ITGB4 interaction occurs at their C-terminals. **a** GST or GST-TMEM268 fusion protein were immobilized on glutathione-Sepharose beads and incubated with BGC823 cell lysates at 4 °C for 4 h. ITGB4 and GST were detected in the washed beads by western blotting. **b** Total BGC823 cell extracts were subjected to IP using either an anti-TMEM268 antibody or an IgG isotype control. ITGB4 was detected in the immunoprecipitates by western blotting. **c**, **d** Construction of truncated TMEM268 and ITGB4 plasmids. **e** BGC823 cells were transfected with FLAG-TMEM268, FLAG-TMEM268-N, or FLAG-TMEM268-C for 24 h. Total cell extracts were subjected to IP using either an anti-FLAG antibody or IgG isotype control. ITGB4 and FLAG were detected in the immunoprecipitates by western blotting. **f** GST and GST-TMEM268, GST-TMEM268-N or GST-TMEM268-C fusion protein immobilized on glutathione-Sepharose beads were incubated with BGC823 cell lysates. ITGB4 and GST were detected in the washed beads by western blotting. **g** 293T cells were cotransfected with FLAG-TMEM268 and either HA-ITGB4-C1, HA-ITGB4-C2 or HA-ITGB4-C3 for 24 h. Total cell extracts were subjected to IP using an anti-HA antibody. ITGB4 mutants and FLAG-TMEM268 were detected in the immunoprecipitates by western blotting

To identify TMEM268 binding partner, we performed a pull-down in BGC823 cell lysates using GST-tagged full-length TMEM268 protein. We found that GST-TMEM268 fusion protein pulled down endogenous ITGB4 (Fig. 4a). The endogenous co-immunoprecipitation (Co-IP) assay revealed that TMEM268 coprecipitated with ITGB4 (Fig. 4b), indicating TMEM268 and ITGB4 may associate together as a complex.

We next constructed different TMEM268 and ITGB4 mutants (Fig. 4c, d) and examined which domains were responsible for the TMEM268−ITGB4 interaction. Data from the Co-IP experiments suggest that the C-terminal of TMEM268 (TMEM268_104–342_) mediates the TMEM268−ITGB4 association (Fig. 4e). GST pull-down further confirmed the interaction between ITGB4 and GST-TMEM268_104–342_ (Fig. 4f). Further Co-IP assays indicated that the C-terminal of ITGB4 (ITGB4_1356–1756_), which contains Fnlll3 and Fnlll4, interacts with TMEM268.

**Fig. 5:**
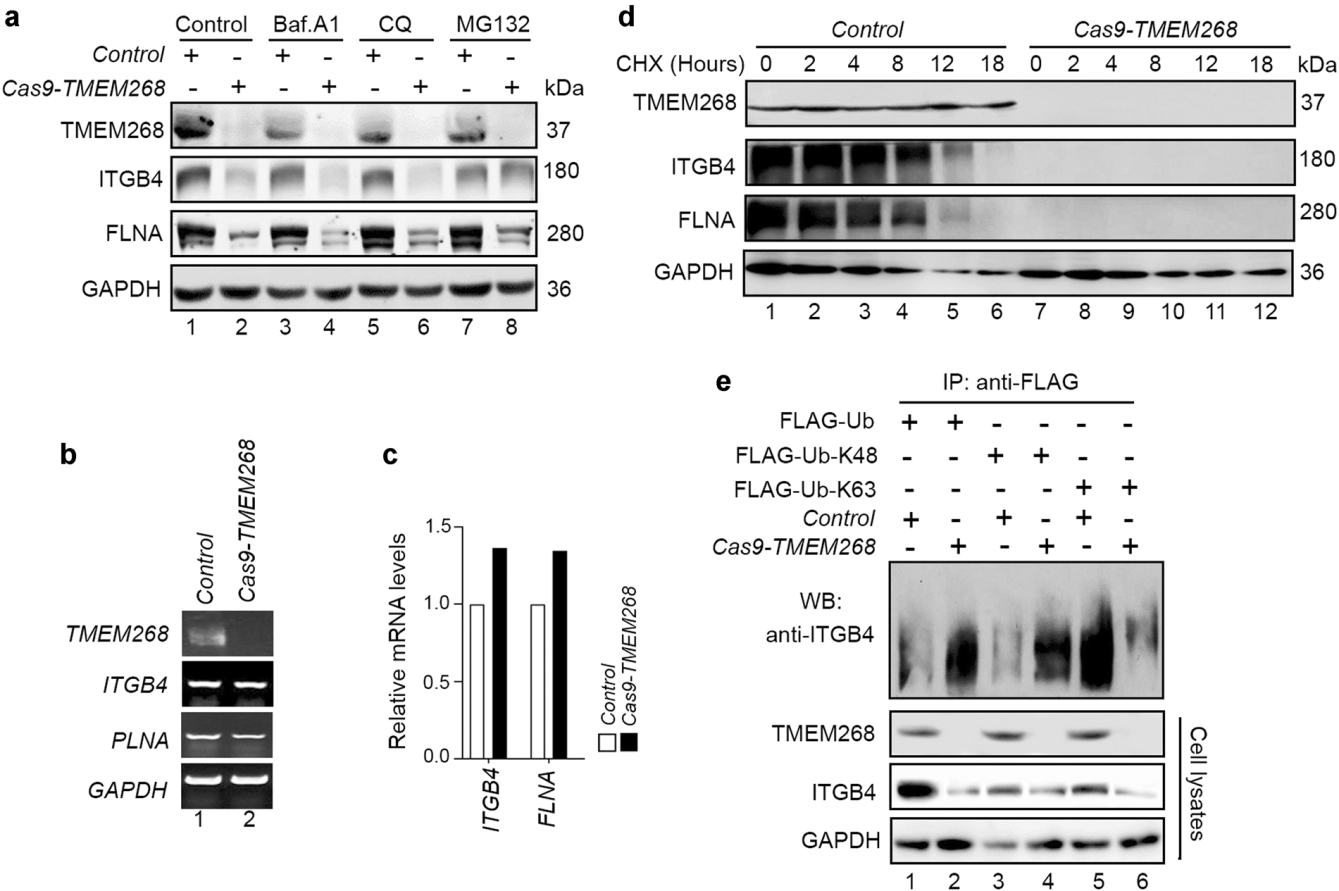
*TMEM268* knockout promotes ITGB4 ubiquitination and degradation. **a** *Control* and *Cas9-TMEM268*/BGC823 cells were treated with different small molecules for different time (10 nM of Baf.A1 or 25 μM of CQ for 4 h; 10 mM MG-132 for 6 h). The cell lysates were then probed with anti-ITGB4 and anti-FLNA antibodies. GAPDH was used as the loading control. **b** *Control* and *Cas9-TMEM268*/BGC823 cells were treated with cycloheximide (CHX, 10 nM) for the indicated times. The levels of endogenous ITGB4 and FLNA were measured by western blotting. **c**, **d** RT-PCR and quantitative real-time RT-PCR were performed to assess mRNA levels of *ITGB4* and *FLNA* in *Cas9-TMEM268*/BGC823 cells. **e** *Control* and *Cas9-TMEM268*/BGC823 cells were transfected with the indicated plasmids for 24 h. The cell lysates were then immunoprecipitated with an anti-FLAG antibody and probed with an anti-ITGB4 antibody

We next explored the biochemical consequence of the TMEM268−ITGB4 interaction. In *Cas9-TMEM268/*BGC823 cells, the levels of ITGB4 are significantly decreased (Fig. 5a, lane 2 vs. lane 1), consistent with the results of the immunofluorescence assay (Fig. 3b). These data indicate that TMEM268 may mediate the stability of ITGB4. *TMEM268* deletion also downregulates the expression of FLNA (Fig. 5a, lane 2 vs. lane 1). Notably, the mRNA levels of *ITGB4* and *FLNA* obtained from RT-PCR and quantitative RT-PCR assays remain unchanged in *TMEM268*-deleted cells (Fig. 5b, c), suggesting that TMEM268 may regulate the post-translational modification of ITGB4.

We next investigated whether ITGB4 protein stability is related to proteasomal or lysosomal degradation. We added the proteasome inhibitor MG-132 (10 μM), or the autophagic inhibitors chloroquine (CQ) or bafilomycin A1 (Baf.A1) which block autophagosome fusion with lysosomes, to *Cas9-TMEM268/*BGC823 or *Control* cells. The results show that ITGB4 degradation in *Cas9-TMEM268/*BGC823 cells is significantly blocked after treatment with MG-132 (Fig. 5a, lane 8 vs. lane 7), but not CQ or Baf.A1 **(**Fig. 5a, lane 6 vs. lane 5, lane 4 vs. lane 3), demonstrating that deletion of *TMEM268* leads to increased proteasomal degradation of ITGB4. We next measured the half-life of ITGB4 using the protein translation inhibitor cycloheximide (CHX) in *CAS9-TMEM268/*BGC823 cells. As shown in Fig. 5d, inactivation of *TMEM268* significantly promotes the decay of ITGB4, indicating that TMEM268 may be a positive regulator of ITGB4.

We next investigated whether TMEM268 regulates ITGB4 stability by modulating its ubiquitylation. The results were shown in Fig. 5e (lane 2 vs. lane 1), total polyubiquitylated ITGB4 protein is significantly increased in *Cas9-TMEM268/*BGC823 cells. Interestingly, *TMEM268*-deleted cells exhibit an increase in K48-linked (Fig. 5e, lane 4 vs. lane 3**)** and a decrease in K63-linked ubiquitylation (Fig. 5e, lane 6 vs. lane 5) in the ITGB4 immunoprecipitates. This result suggests that TMEM268 may prevent K48-linked but facilitate K63-linked polyubiquitination of ITGB4. Combined with the results of Fig. 3b, that showed decreased ITGB4 fluorescent signaling in *Cas9-TMEM268/BGC823* cells, we conclude that TMEM268 may play an important role in maintaining the homeostasis of the ITGB4 protein.

### *TMEM268* deficiency causes cytoskeleton remodeling and cytoskeletal network damage

**Fig. 6:**
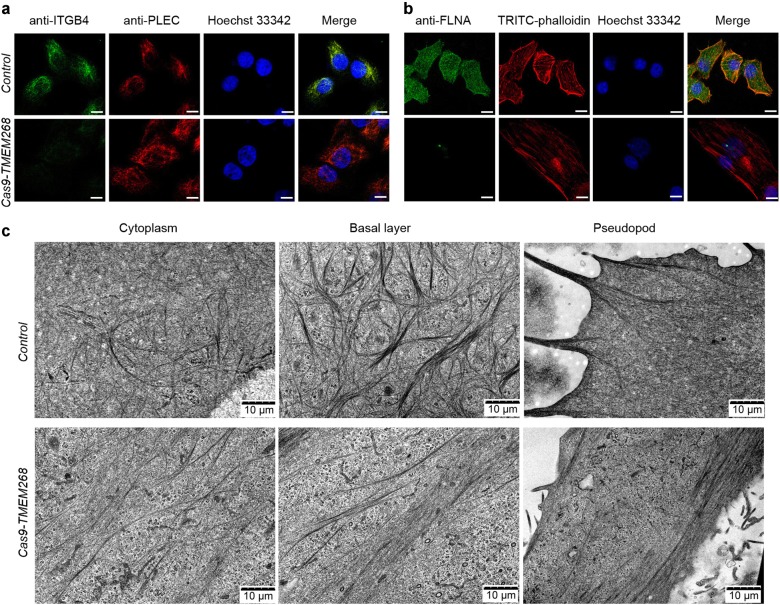
*TMEM268* deficiency causes cytoskeleton remodeling and cytoskeletal network damage. **a**, **b** Control and *Cas9-TMEM268*/BGC823 cells were plated on glass slides for 24 h, then an immunofluorescence assay performed with the indicated antibodies. Nuclei were stained with Hoechst 33342. Representative images obtained from confocal microscopy are shown. Scale bars, 10 μm. **c** Changes in the microfilament network structure in control and *Cas9-TMEM268*/BGC823 cells were observed by transmission electron microscopy. Representative images obtained are shown

It is widely speculated that the association of integrin with the cytoskeleton is necessary to stabilize adhesion to the ECM [26]. PLEC binds ITGB4 and mediates the hemidesmosomal cytoskeleton, which plays an important role in the integrity of the cytoskeleton [27]. In control BGC823 cells, the fluorescence signals of ITGB4 and PLEC showed cluster distribution and complete colocalization (Fig. 6a, upper panel). In *Cas9-TMEM268/*BGC823 cells, the fluorescence signal of ITGB4 was almost undetectable, while PLEC displayed a diffuse filamentous distribution (Fig. 6a, lower panel).

It has been shown that when ITGB4 expression is reduced, dissociated PLEC can bind to intracellular F-actin to maintain the homeostasis of PLEC [28]. Using TRITC-phalloidin staining, we analyzed the distribution of F-actin and the colocalization between PLEC and F-actin in *Cas9-TMEM268/*BGC823 cells. The F-actin microfilament monomer is polymerized into a stable intersecting structure, with no obvious colocalization between PLEC and F-actin in *Control* cells (Fig. S7, upper panel). In *Cas9-TMEM268*/BGC823 cells, F-actin shows a long and sparsely filamentous structure, and the colocalization of PLEC/F-actin significantly increases (Fig. S7, lower panel). The results imply that the ITGB4 degradation induced by *TMEM268* inactivation may directly cause the disintegration and structural damage of hemidesmosomes. Excess PLEC combines with F-actin, and the cytoskeleton is remodeled.

The actin-binding protein FLNA can crosslink F-actin filaments to form a stable cytoskeleton. Western blotting results indicate that the FLNA levels are significantly lower in *Cas9-TMEM268/*BGC823 cells than in *Control* cells (Fig. 5a). Similar to ITGB4, *TMEM268* knockout also promotes the decay of FLNA (Fig. 5d). Results from immunofluorescence assays show that the fluorescence signal of FLNA is almost quenched in *Cas9-TMEM268*/BGC823 cells (Fig. 6b). The colocalization between F-actin and FLNA, therefore, failed to occur (Fig. 6b), indicating that TMEM268 may regulate the stability of FLNA. These results provide further evidence that *TMEM268* knockout affects the reconstruction of the cytoskeleton.

Data from transmission electron microscope analysis demonstrated that actin filaments show cross-linked architecture in the cytoplasm and pyknotic network structure in the basal layer in *Control* cells (Fig. 6c, upper panel). These three-dimensional structures possess high strength, which can support cell pseudopodium extension. In *Cas9-TMEM268/*BGC823 cells, the three-dimensional reticular architecture of actin filaments is destroyed, and instead the filaments are constructed into a compact bundle presenting a long and straight filamentous structure. These parallel microfilaments affect the formation and extension of pseudopodia (Fig. 6c, lower panel), resulting in the destruction of the cytoskeleton. Collectively, we have demonstrated that TMEM268 binds to ITGB4, and may act as an adaptor between ITGB4 and microfilament skeleton remodeling.

### *TMEM268-*deleted cells failed to colonize the lungs after intravenous injection and to form metastatic engraftment in the peritoneum

Since *TMEM268* inactivation impairs the adhesion and growth of gastric cancer cells, we therefore assessed whether *TMEM268*-deleted BGC823 cells lose the ability for distant metastasis.

**Fig. 7:**
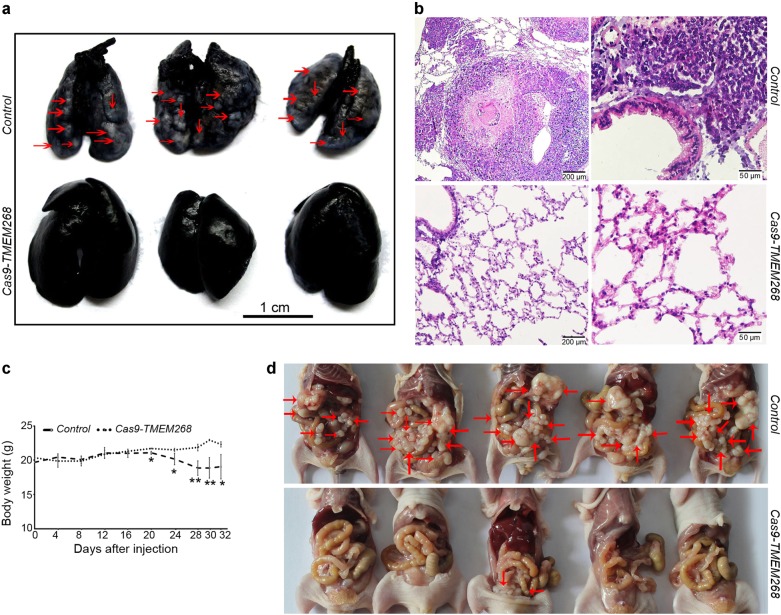
*TMEM268* deletion prevents the metastasis of BGC823 cells. **a** *Control* or *Cas9-TMEM268/*BGC823 cells (1×10^6^ cells/mouse) were injected into BALB/c nude mice (*n* = 6) via the tail vein. On day 54, the mice from each group were sacrificed, and the lungs filled with India ink via the upper trachea and fixed. Metastatic lesions (red arrows) on the black lung surface were observed and photographed. **b** H&E staining of lung tissues. **c** *Control* or *Cas9-TMEM268/*BGC823 cells (8×10^6^ cells/mouse) were injected into the abdominal cavity of mouse (*n* = 6). The changes in body weight were monitored every 4 days after injection (*P < 0.05; **P < 0.01). **d** Images of the peritoneal metastatic nodules were obtained at day 32. Arrows indicate the disseminated tumor nodules.

*Cas9-TMEM268/*BGC823 cells and *Control* cells were injected into nude mice via the tail vein. On day 54, the mice from each group were sacrificed. The lungs were filled with India ink via the upper trachea and fixed. Metastatic nodules on the black lung surface were observed and stained with hematoxylin and eosin (H&E). As shown in Fig. 7a, gross examination revealed that many metastatic tumors appear as white nodules on the black lung surfaces in the *Control* group, while no metastatic nodules are observed in the *Cas9-TMEM268* group. Data obtained from histological analysis demonstrates that, in the *Control* group, there are many tumor cell masses in the alveoli, the *Cas9-TMEM268* group displays the normal tissue structure of the lungs (Fig. 7b). These results suggest that *TMEM268*-deleted cells failed to colonize the lungs after intravenous injection.

To further confirm the effects of TMEM268 on tumor metastasis, an animal model of peritoneal metastasis was established by the intraperitoneal injection of *Control* or *Cas9-TMEM268*/BGC823 cells into mice. The weight curve revealed that the body weight of the *Control* mice decreased after 20 days, while *Cas9-TMEM268* group was maintained at normal level or slightly upregulated (Fig. 7c). All mice were sacrificed at day 32 after injection. As shown in Fig. 7d, a large number of metastatic nodules were found in the peritoneal cavity in *Control* mice. By contrast, the *Cas9-TMEM268* group had little or no tumor nodules. These data suggested that inactivation of *TMEM268* might prevent peritoneal homing and growth of metastatic nodules in gastric cancer cells.

## Discussion

*TMEM268*, a novel human gene associated with cell viability was first identified by our group. It is evolutionarily conserved and widely expressed in many cell lines and tissues. Our studies have demonstrated that inactivation of *TMEM268* can inhibit cell growth, reduce cell adhesion, induce cell cycle arrest, and disrupt cytoskeleton reorganization. Subsequently as a result, the abilities of tumorigenicity and metastasis were significantly decreased (Fig. S8).

Integrin is a complex composed of α and β subunits that associate by noncovalent binding. The different heterodimeric forms can bind different ligands and mediate a variety of cellular activities [29]. The subunits ITGB4 and ITGA6 can bind specifically and form the LN receptor. This complex is involved in cell−ECM and cell−cell adhesion. By integrating the intracellular domain of ITGB4 and its downstream signaling molecules, the cells can react efficiently and accurately to external stimuli. ITGB4 and PLEC form the main structure of hemidesmosomes, which are important structures for epithelial cell adherence to the ECM and for protection of cells from external mechanical stress [30, 31]. Our study found that the loss of *TMEM268* caused ITGB4 polyubiquitination and degradation, as well as reduced ITGA6 levels. Downregulation or disappearance of the ITGA6-ITGB4 complex directly decreases the adhesion ability of *TMEM268* knockout cells and LN. Simultaneously, downregulated ITGA1, ITGAL1, and ITGAV adhesion molecules were also found in *TMEM268* knockout cells, which resulted in a decrease in cell−cell and cell−ECM adhesion. Subsequently, this leads to cells detaching from the bottom of culture plates. Our results suggest that TMEM268 may be a new membrane stability molecule, which plays an important regulatory role in the homeostasis and quality control of adhesion molecules.

The main structure of hemidesmosomes is composed of PLEC and ITGB4. PLEC is an important part of the cytoskeleton, and it is widely expressed in various types of cells [32]. PLEC contains a conserved actin-binding domain, which interacts with F-actin and respond to external stimuli [28]. In a normal physiological state, TMEM268 binds with the ITGB4-PLEC complex and may maintain hemidesmosome formation into three-dimensional filamentous structures. In cells lacking TMEM268, the reduced levels of ITGB4 protein results in the disassociation of the ITGB4/PLEC complex. Dissociated PLEC binds to F-actin and forms a long bunchy structure. Additionally, reduced FLNA makes actin F-actin unable to form a vertical branching structure. Such effects can lead to the disorder of the three-dimensional structure of the cytoskeleton microfilaments and damage the cytoskeleton network structure [33–35]. The detailed mechanism of TMEM268 affecting cytoskeleton remodeling needs further investigation.

Metastasis is a complicated biological cascade that starts with local invasion by tumor cells and continues with migration to distant tissues and ultimately colonization [36]. Among the cancer cells that arrive at a metastatic target organ, only the cells that have acquired the capacities of adhesion, migration, invasion, and proliferation can form a new tumor focus. Increasing evidence demonstrates that extracellular matrix protein 1 promotes cell metastasis by interacting with ITGB4 and inducing ITGB4/FAK signal pathway [17]. ITGB4-mediated invasion and migration of gastric cancer cells is also regulated by metallopanstimulin-1 [18]. Data from in vitro and animal model experiments suggest that TMEM268 interacts with ITGB4, and its levels are greatly depressed in *TMEM268*-deleted cells via proteasome degradation pathway. Such effect thereby leads to a decrease in cell adhesion, survival, and proliferation of *TMEM268-*inactivated cells. Obviously, these circulating tumor cells in the blood may lose the ability to adhere to the vascular endothelium, fail to extravasate into the new tissue (such as lung) and eventually deprives metastatic colonization. The above results provide a new perspective that targeting the TMEM268/ITGB4 signaling axis may be effective in the treatment of metastasis in gastric cancer, which requires further investigation.

In conclusion, we demonstrate that TMEM268 is a positive regulator of ITGB4. *TMEM268* deficiency in gastric cancer cells inhibits adhesion, decreases cell proliferation, regulates cytoskeleton remodeling, and finally depresses tumorigenesis and metastasis. Further studies will focus on collaborating with the clinical laboratory to explore the relationship between TMEM268 levels and clinicopathological parameters of gastric cancer, which will offer evidence for estimating the possibility of TMEM268 as a potential target to prevent/treat gastric cancer.

## Materials and methods

### Antibodies and reagents

Polyclonal or monoclonal antibodies were as follows: anti-TMEM268(Sigma-Aldrich, HPA017199), anti-PLEC (Abcam, ab11220); anti-ITGB4 (14803), anti-FLNA (4762), and anti-ubiquitin (3936) from Cell Signaling Technology; anti-GAPDH (KM9002), anti-GST (KM8005), anti-FLAG (KM8002L), anti-GFP (KM8009L) from Tianjin Sungene. These antibodies conjugated with FITC were purchased from Biolegend: anti-ITGA1 (328307), anti-ITGA6 (313605), anti-ITGAL (301206), anti-ITGAV (327907), and anti-ITGB4 (327805). Following antibodies were from Santa Cruz Biotechnology: anti-CCNE1 (sc-377100), anti-CDKN1B (sc-1641), anti-CCKN1C (sc-56341), anti-SKP2 (sc-74477), anti-CCNB1 (sc-166210), anti-CDK1 (sc-54). Secondary antibodies included DyLight 800/DyLight 680-conjugated IgG against mouse (Rockland, 610-145-002/610-144-002) or rabbit (Rockland, 611-145-002/611-144-002) and FITC/RBITC-conjugated IgG against mouse (Bioss Inc., bs-0296GFITC/bs-0296G-RBITC) or rabbit (Bioss Inc., bs-0295G-FITC/bs-0295G-RBITC). Other reagents used in this study were from Sigma-Aldrich: bafilomycin A1 (BafA1, B1793), MG-132 (C2211), Hoechst 33342 (14533), TRITC-phalloidin (P1951). Cycloheximide (CHX, 239763) was from Calbiochem.

### Plasmid construction

The *TMEM268* cDNA was amplified from BGC823 cells by PCR using the forward primer (5′-CCGGAATTCATGGCCTgTgAACCACAGGTG-3′) and reverse primer (5′- CGGGATCCGCGCTCACCTCGCCAGGAACGG-3′). The insert was released by *Eco*RI and *Bam*HI, then subcloned into the *Eco*RI site of pcDNA.3.1/myc-His(-)B (Life Technologies, V85520) to construct the pcDB-TMEM268 plasmid. Based on this plasmid, we constructed the following plasmids in succession: GFP-TMEM268, Flag-TMEM268, GST-tagged TMEM268 and mutants (Fig. 4c). Different GFP-ITGB4 mutants (Fig. 4d) were also constructed in our laboratory. All constructs were confirmed by DNA sequencing. Specific shRNA mediating *TMEM268* gene knockdown with the targeting sequence, 5′- CACCGCCAGTTGTGTGTTGTCATTTCAAGAGAATGACAACACACAACTGGCTTTTTTG-3′, and nonsilencing shRNA vector were constructed by ORIGEN Corporation. The shRNA vector sequence with no sequence homology to any known human gene was used as the control.

### Cell culture, transfections, and treatments

BGC823, SGC7901, and HEK293 cell lines were cultured in Dulbecco's Modification of Eagle's Medium (DMEM) (Invitrogen, 12800-017) supplemented with 10% Ausbian fetal calf serum (FCS) (Shanghai VIAN-SAGA Biotech. Ltd, Shanghai, China). Cells were transfected with plasmids using NEOFECT Reagent (Neofect, Beijing, TF201201), according to the manufacturer’s instruction, while shRNA transfection was performed using Lipofectamine 3000 reagent (Life Technologies, L3000-015).

### Knockout of the *TMEM268* gene by CRISPR/Cas9-mediated genome editing

*TMEM268* knockout cell line by CRISPR/Cas9-mediated genome editing was established in BGC823 cells. Target sequences for CRISPR interference were designed by Shanghai Biomodel Organism Science & Technology Development Co., Ltd (China). The target sequences for human *TMEM268* are CAATGGCCAGGTCCTCACTG (Exon 2). The *TMEM268* DNA was amplified by PCR using the forward primer (5′- TCTCACATTGTGGTCCTAGGA-3′) and reverse primer (5′- TACGACAGCTGGTACAGA-3′). Sequence analysis revealed that the mutation (ACAATG → TG) resulted in frameshifts with premature termination by introducing stop codon, removal of translation initiation codon and deletion of functional domain such as TM domain of TMEM268.

### Reverse transcription PCR and quantitative real-time PCR assays

Total RNA samples were extracted from cells with the TRIzol reagent (Invitrogen, 15596-026). RT-PCR was performed using the ThermoScript RT-PCR System (Invitrogen, 11146-024). Quantitative real-time PCR was performed using SYBR Premix Ex Taq (TaKaRa, Japan). The primers against the indicated genes used in this study were listed in Supplementary Table 1.

### Immunofluorescence, fluorescence, and confocal microscopy assays

Cells were cultured in confocal dishes and treated as indicated, fixed with 4% paraformaldehyde and permeabilized with 0.2% Triton X-100. The dishes were then incubated with fetal bovine serum overnight and exposed to primary antibodies for 1 h at 4 °C. After washing three times with phosphate-buffered saline (PBS), the dishes were covered with FITC/TRITC-conjugated secondary antibodies. Nuclei were stained with Hoechst 33342. Morphological alterations in the cells were observed and documented using an Olympus FluoViewTM FV1000 Confocal Microscope (Olympus, Melville, NY, USA).

### Transmission electron microscopy assays

Treated BGC823 cells were fixed in 0.1 M sodium phosphate buffer containing 2.5% glutaraldehyde (pH 7.4) for 1 h at 37 °C. Cells were embedded in Ultracut (LEICA ULTRACUT R, Bensheim, Germany) and sliced into 70-nm sections. Ultrathin sections were stained with uranyl acetate and lead citrate, and observed under a HT7700 transmission electron microscope (HITACHI-JPN, Ltd.).

### Cell viability assay

Control and *Cas9-TMEM268*/BGC823 cells were treated with serum deprivation for 24 h. Then, cells were cultured with the complete culture medium for indicated times. Cell viability assays were performed using the CellTiter 96 AQueous One Solution Cell Proliferation Assay (Promega, G1111) according to the manufacturer’s instructions. The absorbance was measured on an EL-311SX ELISA Reader (Bio-Tec Instruments, USA) at 490 nm. Cell viability was calculated as follows: cell viability = absorbance of test group/absorbance of control cell group × 100%. Each experiment was performed in biological triplicate and independently repeated four times.

### Clone formation assay

Cells were plated in triplicate at 200 cells/well in 24-well plates and cultured for 10−14 days. Cells were then fixed with 4% paraformaldehyde for 30 min and stained with crystal violet for 30 min. Colonies containing more than 50 cells were counted and photographed. All of the experiments were repeated three times, and the average values were reported.

### Cell cycle analysis

Control and *Cas9-TMEM268/*BGC823 cells were treated with serum deprivation for 24 h. Then, cells were cultured with the complete culture medium for indicated times. The cells were fixed with 70% ethanol at −20 °C overnight, then treated with 100 μg/ml RNase A for 30 min at 37 °C, and stained using PI in 0.2% Triton X-100. Subsequently, the cells were collected on an FACS Calibur flow cytometer and the percentages of cells in the phases of the cell cycle (G0/G1, S and G2/M) were analyzed by ModFit software (BD Biosciences, Franklin Lake, NJ, USA).

### Cell−matrix adhesion assays

Ninety-six-well plates were coated with 5 μg/ml plasma fibronectin (Sigma, F1141) or 5 μg/ml laminin (Corning, 354232) overnight at 4 °C. Subsequently, nonspecific binding sites were blocked with 1% bovine serum albumin (BSA) in PBS for 1 h, then plates were washed by PBS. The cells (4×10^4^ cells/100 μl) diluted with DMEM were added to the coated 96-well plates and incubated at 37 °C for 30 min. Nonadherent cells were removed by washing with DMEM. Attached cells were analyzed using MTS according to the manufacturer’s instructions, and the optical density was measured at 490 nm. These experiments were performed in six duplicate and repeated three times.

### GST affinity isolation assay

Recombinant GST, GST-TMEM268, or GST-TMEM268 mutants were expressed in *Escherichia coli* strain BL21 (DE3) and purified. Equal amounts of these proteins immobilized on glutathione-Sepharose TM 4B (GE Healthcare, 17-0756-01) were incubated with whole cell lysates extracted from transfected BGC823 cells at 4 °C for 4 h. The beads were then washed and resuspended in 2× sodium dodecyl sulfate (SDS) loading buffer and analyzed by western blot.

### Immunoprecipitation and western blot analysis

For the IP analysis, treated cells were collected and disrupted in IP analysis buffer (300 mM NaCl, 50 mM Tris, pH 8.0, 0.4% NP-40, 10 mM MgCl_2_, 2.5 mM CaCl_2_) containing protease inhibitors (Roche Diagnostics, 04693116001). Total cell extracts (1 mg per sample) were mixed with precleared protein G Sepharose^TM^ Fast Flow (GE Healthcare, 17-0618-01) and appropriate antibodies, followed by incubation for 4 h at 4 °C. The beads were collected by centrifugation, washed five times and resuspended in 2× SDS loading buffer and then subjected to western blotting. The protein bands were visualized using DyLight 800/DyLight 680-conjugated secondary antibodies, and the infrared fluorescence image was obtained using an Odyssey infrared imaging system (LI-CORBiosciences, Lincoln, NE, USA).

### Tumorigenicity in nude mice

A nude mouse xenograft model was established using 6−8-week-old male BALB/c nude mice (Experimental Animal Center, Peking University Health Sciences Center, Beijing, China). Mice were housed and maintained in a pathogen-free facility, and all experimental procedures were approved by the Institutional Authority for Laboratory Animal Care of Peking University. *Control* or *Cas9-TMEM268/BGC823* cells were subcutaneously injected in the right axilla of mice (4×10^6^ cells/mouse, *n* = 6). Mice were sacrificed at 19 days after cell inoculation. Tumors were excised and photographed.

### Long-term lung colonization assay in nude mice

*Control* or *Cas9-TMEM268/BGC823* cells (1×10^6^ cells/mouse) were injected into BALB/c nude mice (*n* = 6) through tail vein. At 53 days after inoculation, the mice were sacrificed. The lungs were filled with 15% India ink via the upper trachea and fixed with Fekete’s solution, then photographed. Some specimen were embedded in paraffin, sectioned, and subjected to H&E staining.

### Peritoneal metastasis assay in nude mice

*Control* or *Cas9-TMEM268*/BGC823 cells (8×10^6^ cells/mouse) were injected intra-peritoneum in BALB/c nude mice (*n* = 6). The changes in body weight were monitored every 4 days after injection. At 32 days after inoculation, the mice were sacrificed and the formation of metastatic nodules was observed and photographed.

### Statistical analysis

Data are presented as the mean ± SD. Differences between groups were analyzed using the Student’s *t* test for continuous variables. Statistical significance in this study was set at *P* < 0.05. All analyses were performed using GraphPad Prism 7.

## Electronic supplementary material

Supplemental data

Supplemental Table 1

Figure S1

Figure S2

Figure S3

Figure S4

Figure S6

Figure S7

Figure S8

Figure S9

Figure S10
