# Supplementary material for: Deletion of TMEM268 inhibits growth of gastric cancer cells by downregulating the ITGB4 signaling pathway
Source: Cell Death Differ. 2018 Oct 25;26(8):1453–66. doi: 10.1038/s41418-018-0223-3 (PMC6748091; doi:10.1038/s41418-018-0223-3)
Supplement: Supplementary file 1 — Supplemental data [file 41418_2018_223_MOESM1_ESM.docx]

**Supplementary Materials and Methods**

**EdU incorporation assay**

Cell proliferation was detected using the EdU detection kit (C10639; Invitrogen) according to the manufacturer’s protocol. Briefly, *shControl* and *shTMEM268/*BGC823 cells were plated on coverslips and EdU was added to the medium 4h before harvesting cells. Cells were fixed in 4% paraformaldehyde and nuclei were counterstained with Hoechst 33342, then observed and documented using an Olympus FluoViewTM FV1000 Microscope (Olympus, Melville, NY, USA).

**Cell apoptosis assay**

*Control* and *Cas9-TMEM268*/BGC823 cells were serum-starved for 18 h and then pulsed with 10% FCS for different time. Cell apoptosis was detected using an FITC-Annexin V/PI staining detection kit (Beijing Biosea Biotechnology Co., Ltd., China) according to the manufacturer’s instruction. Fluorescence signals were detected through a FACSCalibur flow cytometer (BD Biosciences, USA) to determine the percentage of apoptotic cells, which included Annexin V^+^PI^+^ double and Annexin V^+^ single positive cells.

**Transwell assay**

*In vitro* migration assay were carried using 8-μm transwell inserts. *Control* and *Cas9-TMEM268*/BGC823 cells (1 × 10^5^) were incubated for 12 h in serum-free DMEM media at the top chamber of each transwell insert, and serum-containing media was added to the lower chamber. After 24 h, cells that migrated were fixed in 10% formalin, stained with 1% crystal violet, and counted under a light microscope. At same time, cells that in well bottom and in suspension were also counted.

**Supplementary figure legends**

**Supplementary figure 1** Expression profile of human TMEM268. (a) *TMEM268* mRNA expression was analyzed by RT-PCR in human cell lines. *GAPDH* expression was amplified as an internal control. (b) Protein expression of PHF23 in mammalian cell lines was detected by a rabbit anti-TMEM268 antibody using western blot. GAPDH was used as the loading control.

**Supplementary figure 2** Localization of human TMEM268. HEK293 cells were transiently transfected with GFP-TMEM268 and dsRed-ER. 24 h after transfection, cells were stained with Hoechst 33342 (Blue) and confocal analysis was performed.

**Supplementary figure 3** Knockdown of *TMEM268* inhibited cell growth. (a) BGC823 and SGC7901 cells were transfected with either *shTMEM268* or *shControl*, for 24 h. TMEM268 mRNA and protein levels were detected by RT-PCR and western blot, respectively. (b and c) BGC823 and SGC7901 cells were transfected with either *shTMEM268* or *shControl* for different time. Cell viability was detected by MTS assay. (d) BGC823 cells plated in glass slides were transfected with either *shTMEM268* or *shControl* for 48 h and incorporated with EdU for 4 h. Then the indicated cells were performed by immunofluorescence assay. Nuclei were stained with Hoechst 33342. Scale bar, 50 μm. (e) Quantification of the percentage of EdU-positive cells from five randomly selected areas from each slide. Each bar represents the mean±S.D. from three independent experiments. (f) Representative images of the colony formation in BGC823 or SGC7901 cells transfected with *shTMEM268* or *shControl*. (g) Number of clones was shown for three independent experiments. Data are expressed as mean ± SD. **P* < 0.05, ***P* < 0.01, ****P* < 0.001.

**Supplementary figure 4** Generation of *TMEM268* knockout BGC823cells by CRISPR/Cas9-mediated genome editing. Sequencing diagrams for *Cas9-TMEM268* knockout clone.

**Supplementary figure 5** Knockout of *TMEM268* in BGC823 cells did not show apoptosis. *Control* and *Cas9-TMEM268*/BGC823 cells were serum-starved for 18 h, and then pulsed with 10% FCS for different time. Apoptosis was measured by FITC–Annexin-V/PI staining and flow cytometry.

**Supplementary figure 6** Knockout of *TMEM268* in BGC823 cells impairs cell adhesion. (a) *Control* and *Cas9-MARCH2*/BGC823 cells were cultured for 12 h in serum-free DMEM medium, then cells were performed for Transwell assay. Photographs were obtained after 24 h of incubation. (b) The percentage of membrane-adhered cells. Data are presented as the mean ± SD of the results from 3 experiments. (c) The percentage of total cells including membrane-adhered cells and the shedding cells. Data are presented as the mean ± SD of the results from 3 experiments. ****P* < 0.001, n.s, not significance.

**Supplementary figure 7** Knockout of *TMEM268* in BGC823 cells affected the changes of PLEC and F-actin. *Control* and *CAS9-TMEM268*/BGC823 cells were plated on glass slides for 24 h, then performed with immunofluorescence assay by indicated antibodies. Nuclei were stained with Hoechst 33342. Representative images obtained from confocal microscopy were shown. Scale bars, 10 μm.

**Supplementary figure 8** A schematic model of the effect of TMEM268 on gastric cancer cells.
