# Supplementary material for: Deletion of TMEM268 inhibits growth of gastric cancer cells by downregulating the ITGB4 signaling pathway
Source: Cell Death Differ. 2018 Oct 25;26(8):1453–66. doi: 10.1038/s41418-018-0223-3 (PMC6748091; doi:10.1038/s41418-018-0223-3)
Supplement: Supplementary file 2 — Supplemental Table 1 [file 41418_2018_223_MOESM2_ESM.docx]

| Supplemental Table 1. Primers used for RT-PCR and qRT-PCR |
| --- |
| \|  \| Forward Primer \| \| Reverse Primer \| \| --- \| --- \| --- \| --- \| \| RT-PCR \|  \| \|  \| \| *TMEM268* \| \| CCggAATTCATggCCTgTgAAC  CACAggTg \| CgggATCCgCgCTCACCTCgCCA  ggAACgg \| \| *ITGB4* \| \| GCAGCTTCCAAATCACAGAGG \| CCAGATCATCGGACATGGAGTT \| \| *FLNA* \| \| CGAGGTCGAGGTTGTGATCC \| GCAGGCACTCGGGTTACAG \| \| *GAPDH* \| \| GACCACAGTCCATGCCATCAC \| TCCACCACCCTGTTGCTGTAG \| \| qRT-PCR \| \|  \|  \| \| *ITGB4* \| \| gCAgCTTCCAAATCACAgAgg \| CCAgATCATCggACATggAgTT \| \| *FLNA* \| \| CgAggTCgAggTTgTgATCC \| gCAggCACTCgggTTACAg \| \| *GAPDH* \| \| ggAgCgAgATCCCTCCAAAAT \| GGCTGTTGTCATACTTCTCATGG \| \|  \| \|  \|  \| |
