# Supplementary figures and images for: Deletion of TMEM268 inhibits growth of gastric cancer cells by downregulating the ITGB4 signaling pathway
Source: Cell Death Differ. 2018 Oct 25;26(8):1453–66. doi: 10.1038/s41418-018-0223-3 (PMC6748091; doi:10.1038/s41418-018-0223-3)

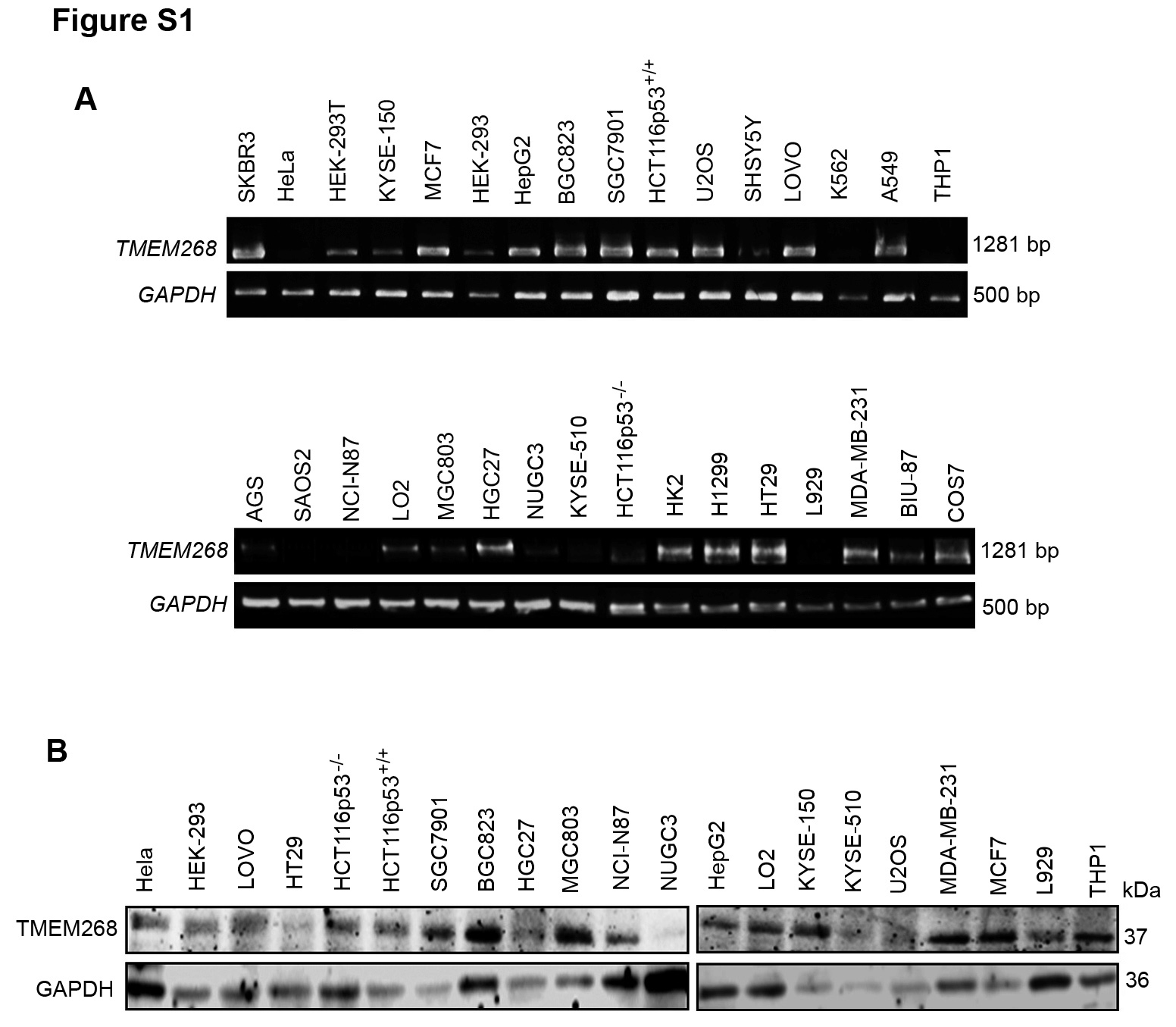

Supplement: Supplementary file 3 — Figure S1 [file 41418_2018_223_MOESM3_ESM.jpg]

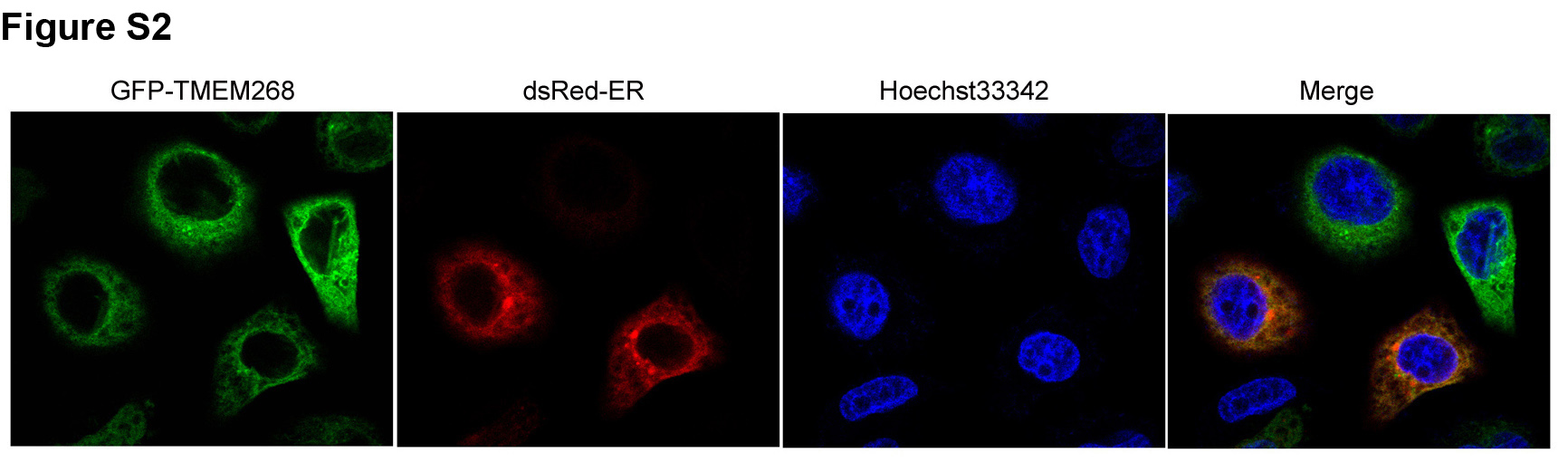

Supplement: Supplementary file 4 — Figure S2 [file 41418_2018_223_MOESM4_ESM.jpg]

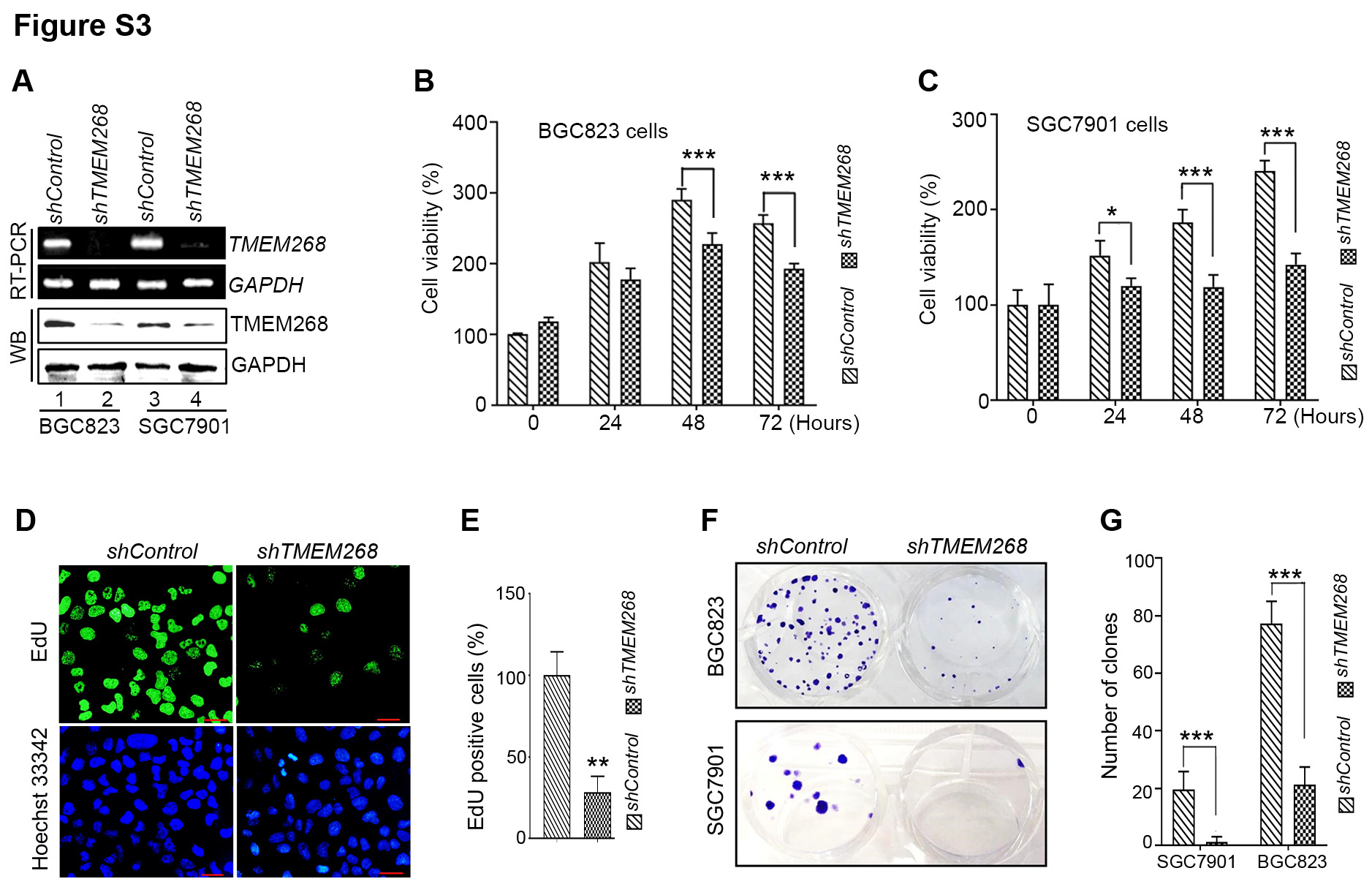

Supplement: Supplementary file 5 — Figure S3 [file 41418_2018_223_MOESM5_ESM.jpg]

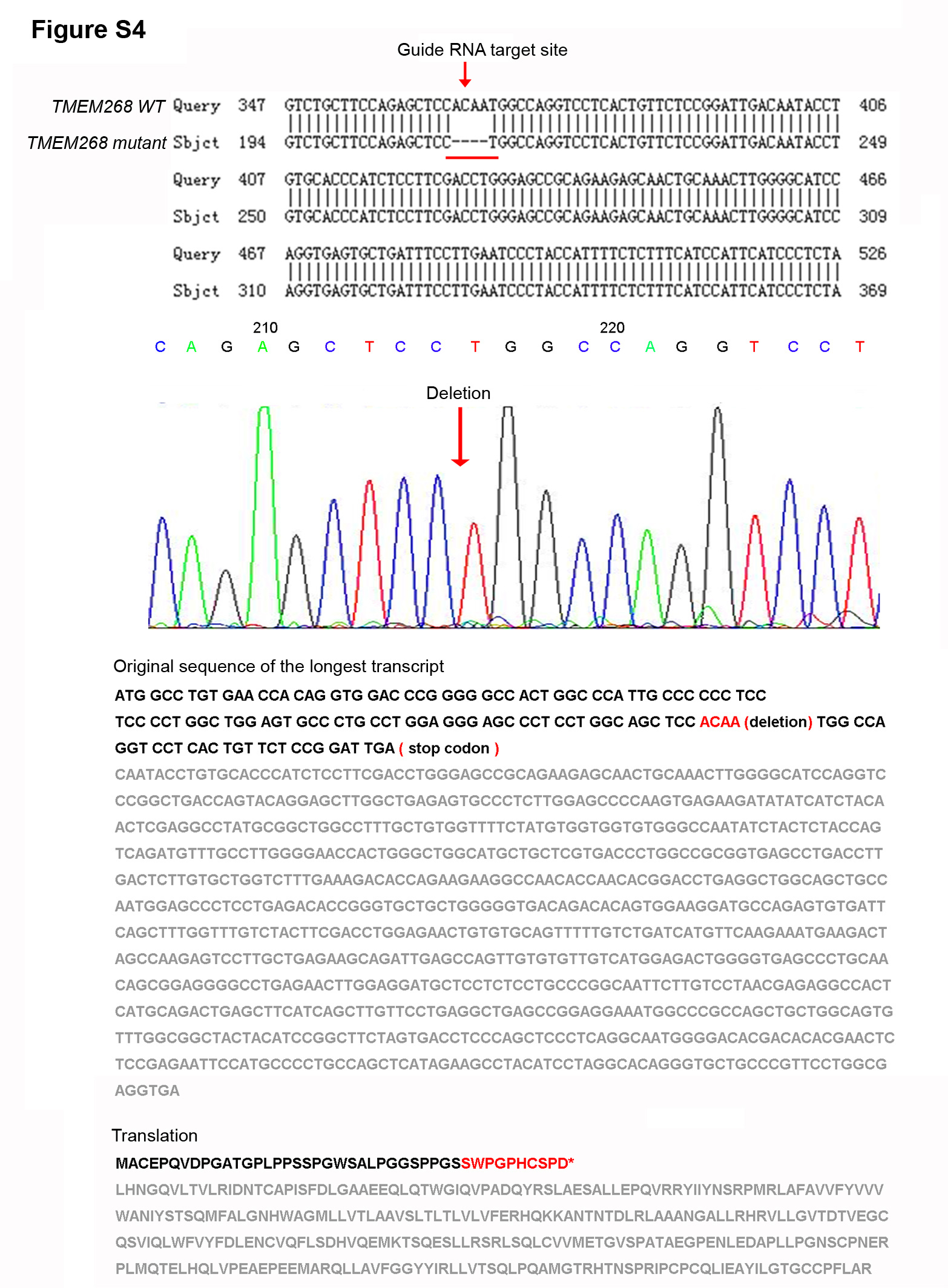

Supplement: Supplementary file 6 — Figure S4 [file 41418_2018_223_MOESM6_ESM.jpg]

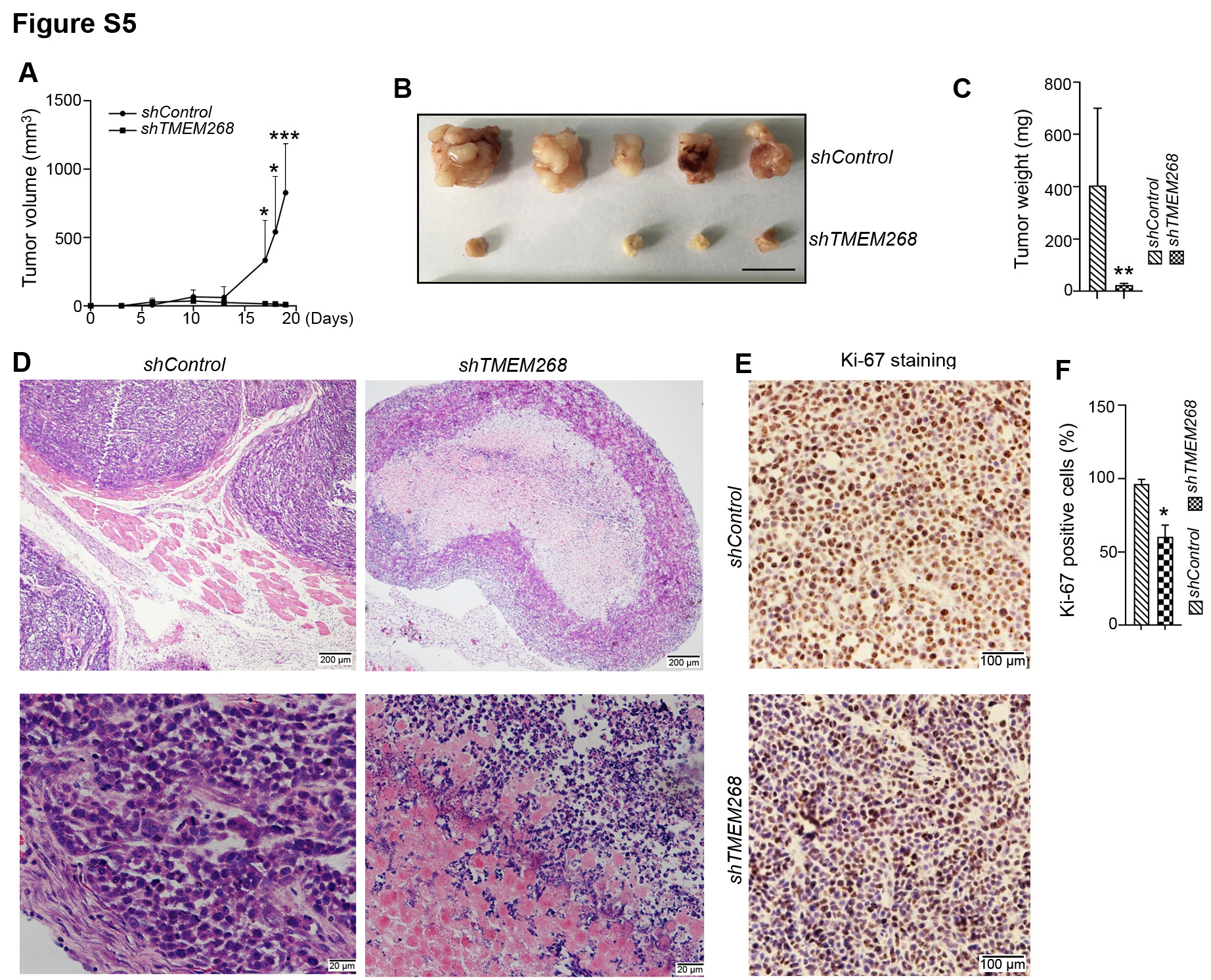

Supplement: Supplementary file 7 — Figure S6 [file 41418_2018_223_MOESM7_ESM.jpg]

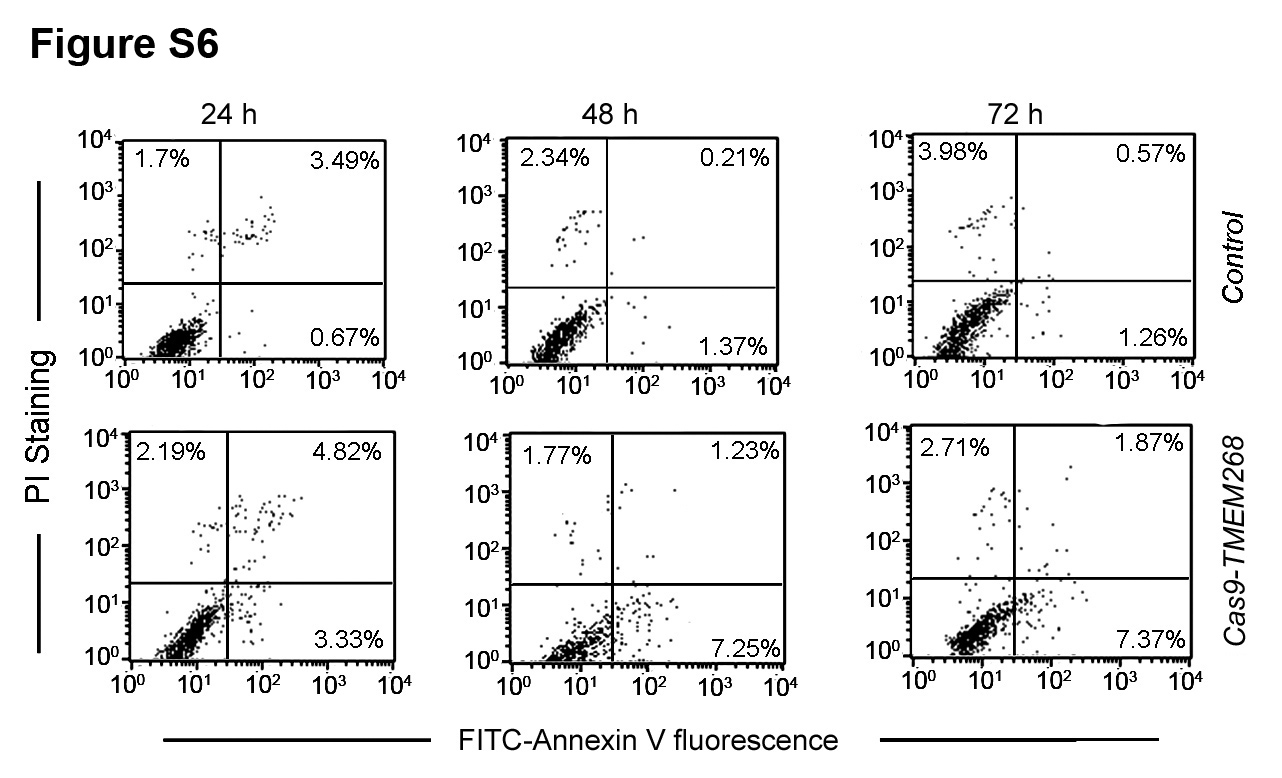

Supplement: Supplementary file 8 — Figure S7 [file 41418_2018_223_MOESM8_ESM.jpg]

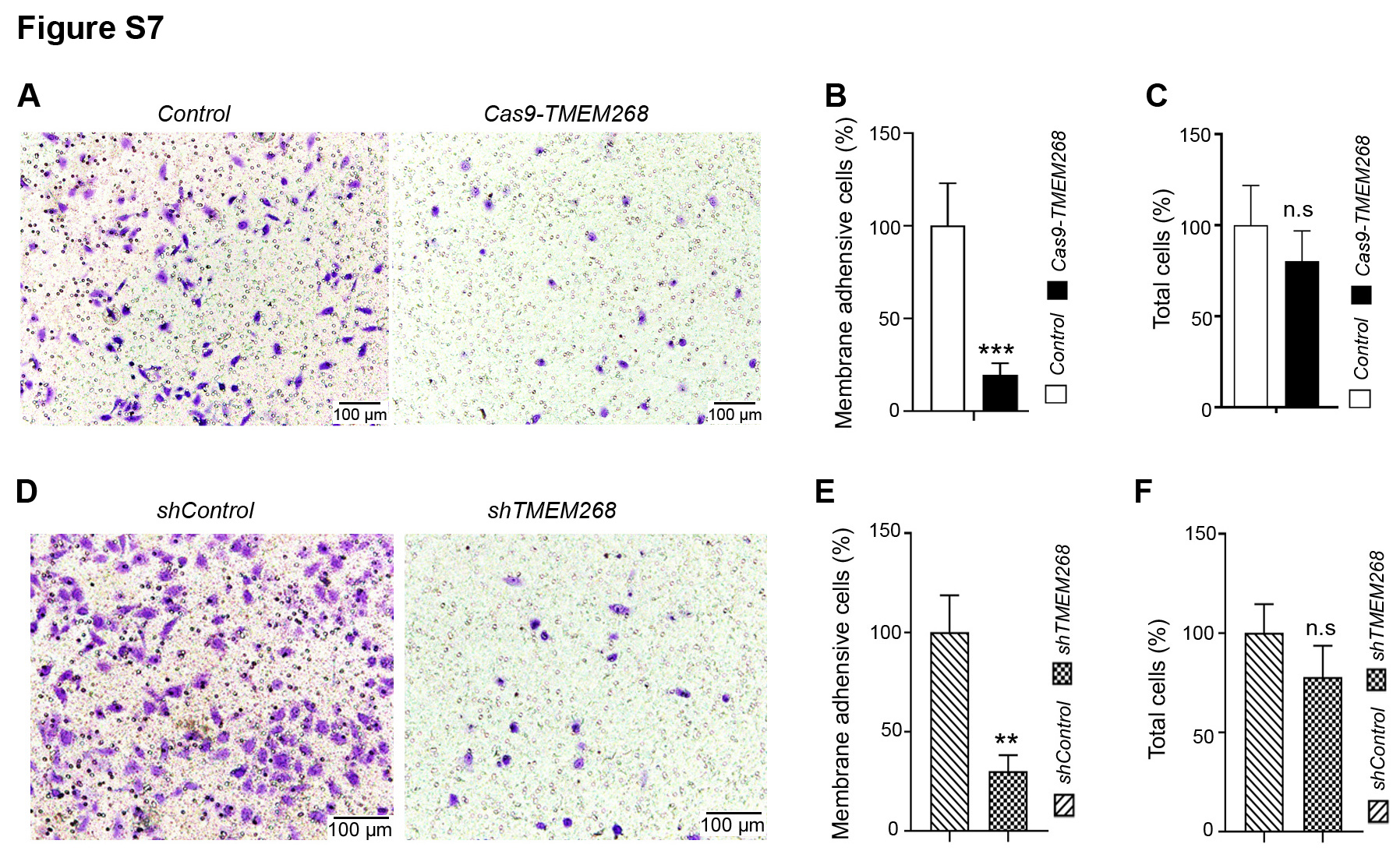

Supplement: Supplementary file 9 — Figure S8 [file 41418_2018_223_MOESM9_ESM.jpg]

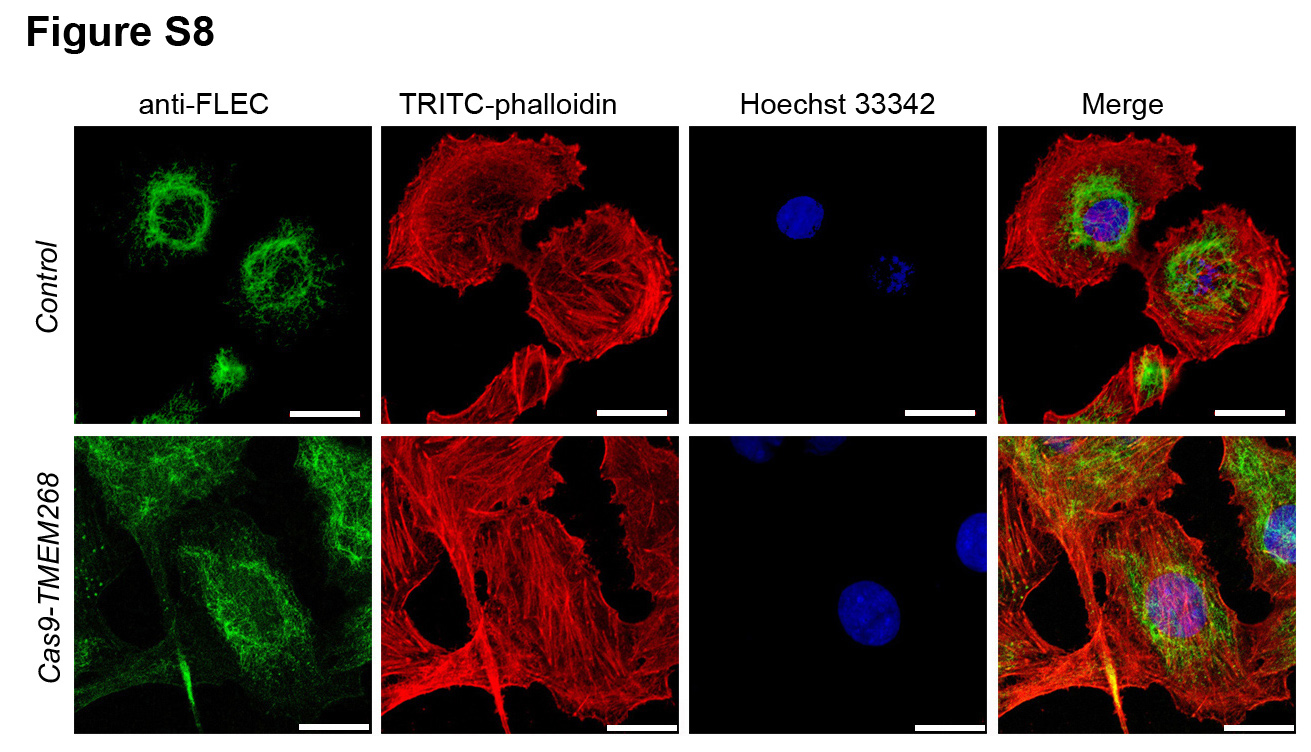

Supplement: Supplementary file 10 — Figure S9 [file 41418_2018_223_MOESM10_ESM.jpg]

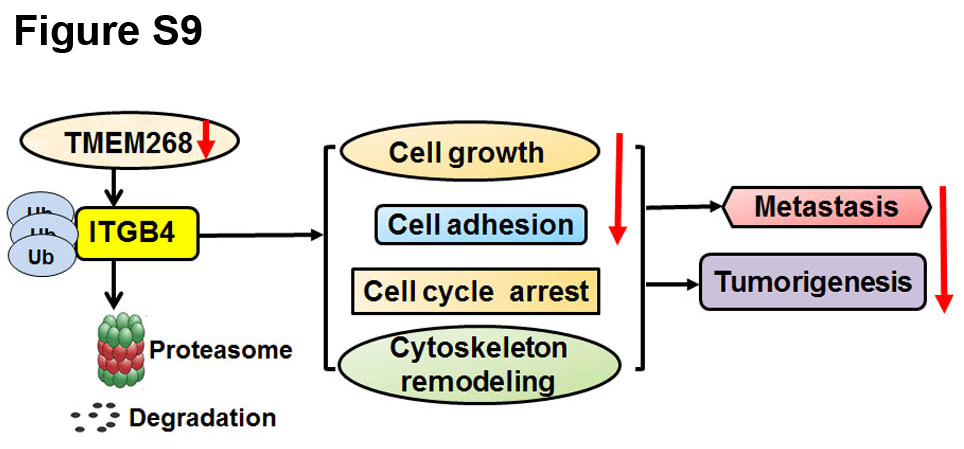

Supplement: Supplementary file 11 — Figure S10 [file 41418_2018_223_MOESM11_ESM.jpg]
